# Structural characterization of a new samarium–sodium heterometallic coordination polymer

**DOI:** 10.1107/S2056989024001051

**Published:** 2024-02-06

**Authors:** Ashley M. Hastings, Ashley Williams, Robert G. Surbella III, Amy E. Hixon, Ana Arteaga

**Affiliations:** a Pacific Northwest National Laboratory, Richland, WA, 99354, USA; b University of Notre Dame, South Bend, IN 46556, USA; Universidad de la República, Uruguay

**Keywords:** crystal structure, samarium, *o*-vanillin, coordination polymer, lanthanide chemistry

## Abstract

Lanthanide-containing materials are of inter­est in the field of crystal engin­eering because of their unique properties and distinct structure types. In this context, a new samarium–sodium heterometallic coordination polymer, poly[tetra­kis­(μ_2_-2-formyl-6-meth­oxy­phenolato)samarium(III)sodium(I)], {[SmNa(C_8_H_7_O_3_)_4_]·solvent}_*n*_ (**Sm-1**), was synthesized and crystallized *via* slow evaporation from a mixture of ethanol and aceto­nitrile. The compound features alternating Sm^III^ and Na^I^ ions, which are linked by *ortho*-vanillin (*o*-vanillin) ligands to form a mono-periodic chain-like coordination polymer. The chains propagate along the [001] direction. Residual electron density of disordered solvent mol­ecules in the void space could not be reasonably modeled, thus the SQUEEZE function was applied. The structural, vibrational, and optical properties are reported.

## Abstract 2

The crystal structure is reported of a new heterometallic samarium compound comprised of alternating Sm^III^ and Na^I^ metal centers bridged by *o*-vanillin ligands to create a helical chain.

## 1. Chemical context

The synthesis of lanthanide compounds with 2-hy­droxy-3-meth­oxy benzaldehyde (*o*-vanillin) ligand derivatives is of great inter­est in the field of crystal engineering because of their photophysical and magnetic properties (Chaudhari *et al.*, 2012 ▸; Song *et al.*, 2017 ▸; Novitchi *et al.*, 2012 ▸; Albrecht, 2001 ▸). In crystal engineering, the ligand of choice has a large effect on the dimensionality of lanthanide-containing compounds owing to their high-coordination environments (Bunzli & Piguet, 2002 ▸). For example, ligands with multiple binding sites are ideal because of their ability to bridge metal centers or act as capping ligands (Heuer-Jungemann *et al.*, 2019 ▸; Cheng & Yang, 2017 ▸). *o*-Vanillin is a popular ligand for heterometallic synthesis due to its ability to generate a variety of compounds through its multiple binding sites (carboxyl­ate and meth­oxy groups; Andruh, 2015 ▸). While there is an extensive library of lanthanide and *o*-vanillin-containing compounds, ranging in dimensionality from small mol­ecules to coordination polymers (CPs) and metal organic frameworks (MOFs) (CSD, version 2021.3.0; Groom *et al.*, 2016 ▸), we are not aware of any reports containing *o*-vanillin, Sm^III^ and Na^I^, and have found only a single report containing both *o*-vanillin and Sm^III^ (Griffiths *et al.*, 2016 ▸). However, heterometallic lanthanide–transition-metal com­pounds with *o*-vanillin have been reported (Costes *et al.*, 2015 ▸, 2018 ▸; Kırpık *et al.*, 2019 ▸). These compounds crystallize as discrete mol­ecular dinuclear units. To the best of our knowledge, the only reported lanthanide–Na^I^–*o*-vanillin-containing compound crystallized as an aggregate structure with a hydro­phobic cavity (Li *et al.*, 2022 ▸). The lanthanide–Na^I^–*o*-vanillin compound isolated by Li *et al.* is vastly different from the structure described here, [SmNa(C_8_H_7_O_3_)_4_]·solvent (**Sm-1**). Herein we report the synthesis, crystal structure, and characterization of an inter­esting new samarium–sodium heterometallic CP synthesized with *o*-vanillin ligands. 
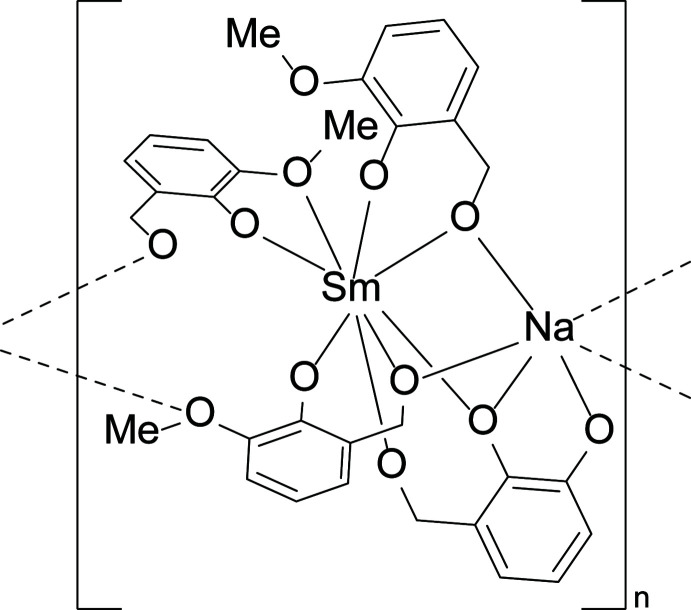


## 2. Structural commentary

**Figure 1:**
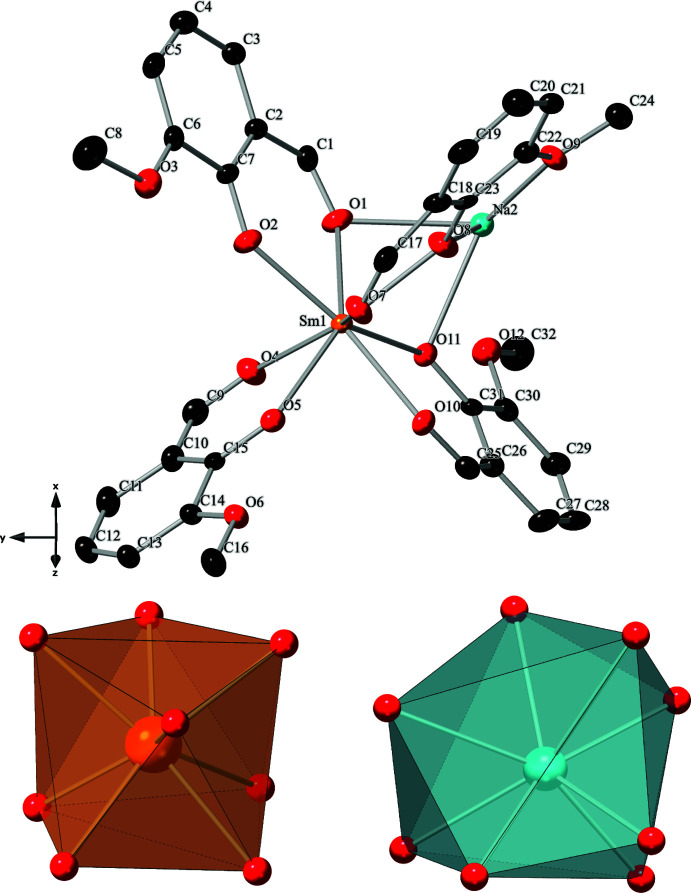
Top: The asymmetric unit of **Sm-1**. The Sm, Na, C, and O atoms are depicted as orange, teal, black, and red ellipsoids, respectively. The displacement ellipsoids are drawn at 50% probability. The hydrogen atoms are removed for clarity. Bottom: The coordination environment of the Sm^III^ and Na^I^ metal centers, represented as orange and teal polyhedra, respectively.

**Figure 2:**
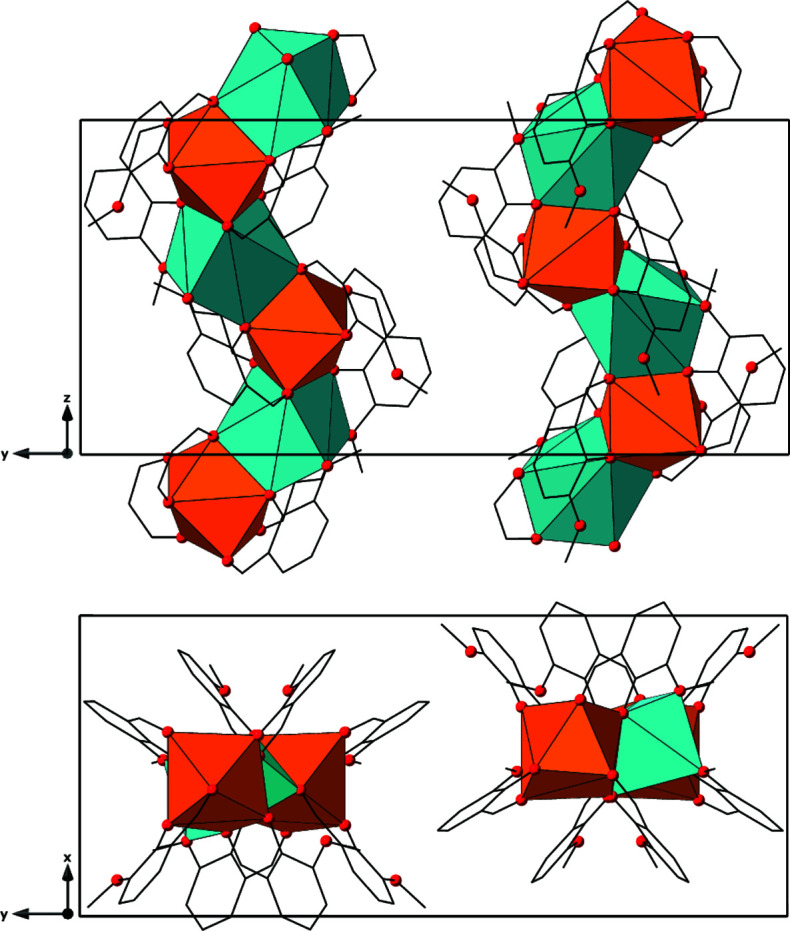
Polyhedral representation of **Sm-1** showing the propagation of the chains along the [001] direction. The Sm^III^ and Na^I^ atoms are represented as orange and teal polyhedra, respectively. The oxygen atoms are represented by red spheres and the carbon atoms are represented in stick form. Hydrogen atoms have been omitted for clarity.

The compound [SmNa(C_8_H_7_O_3_)_4_]·solvent (**Sm-1**) crystallizes in the *P*2_1_/*c* space group. The asymmetric unit features one crystallographically unique Sm^III^ and Na^I^ metal center, and four *o-*vanillin ligands (Fig. 1 ▸). Each metal center is coordinated by eight oxygen atoms, each displaying a distorted square-anti­prismatic geometry with a local *C*_1_ symmetry (Fig. 1 ▸). The Sm^III^ metal centers are bound to four *o*-vanillin ligands (κ^2^) with an average Sm—O bond length of 2.395 (2) Å. The Na^I^ cations are bound to six *o*-vanillin ligands, two of which are bidentate (κ^2^) and four are monodentate (κ^1^), with average Na—O bond lengths of 2.530 (4) Å. The metal-to-oxygen bond distances are typical of those reported in similar systems (Ma *et al.*, 2021 ▸; Peng *et al.*, 2011 ▸). The Sm^III^ and Na^I^ atoms alternate and are bridged together by three μ_2_-*o*-vanillin ligands that each display unique bonding environments through the phenoxo, aldehydic, and meth­oxy groups (see Fig. S1 in the supporting information). The first *o-*vanillin ligand binds the alternating Sm^III^ and Na^I^ atoms through the phenoxo and aldehydic groups, leaving the meth­oxy group uncoordinated, Fig. S1*a*. The second *o-*vanillin ligand bridges the Sm^III^ and Na^I^ atoms using the phenolic group, with the aldehydic and meth­oxy groups binding solely to the Sm^III^ and Na^I^ atoms, respectively, Fig. S1*b*. Lastly, the third *o-*vanillin ligand bridges the alternating Sm^III^ and Na^I^ atoms *via* the aldehydic and phenoxo groups while the meth­oxy group binds solely to an adjacent Na^I^ atom, Fig. S1*c*. This creates a bimetallic helical chain that propagates along the [001] direction (Fig. 2 ▸). The potential solvent area volume of **Sm-1** is 10.6% per unit cell (calculated using *PLATON*; Spek, 2020 ▸).

## 3. Supra­molecular features

The structure was analyzed for non-covalent inter­actions and no evidence for π–π inter­actions was observed. However, a series of close atom contacts (C—H⋯C) are present between adjacent chains (Table 1 ▸). The supra­molecular chains are stabilized primarily through C—H⋯C inter­actions, allowing the stacking of adjacent chains in the structure.

## 4. Database survey

The *o*-vanillin ligand is widely used in coordination chemistry with over 70 structures containing *o*-vanillin and lanthanides reported in the Cambridge Structural Database (CSD, version 2021.3.0; Groom *et al.*, 2016 ▸). A survey of structures containing samarium and *o*-vanillin resulted in only one compound, [Ni_2_Sm_2_(C_14_H_11_NO_3_)_4_(C_8_O_3_H_7_)_2_(H_2_O)_2_]·4CH_3_CN, a heterometallic and heteroleptic cluster containing Sm^III^ and Na^I^ metal centers bound by 2-(*E*)-{[(2-hy­droxy­phen­yl)imino]­meth­yl}-6-meth­oxy­phenol ligands (Griffiths *et al.*, 2016 ▸). In this compound, the *o*-vanillin ligands act as capping ligands and are bidentate (κ^2^) in fashion, whereas in **Sm-1**, the *o*-vanillin ligands act as bridging ligands that connect the Sm^III^ and Na^I^ atoms to form a mono-periodic CP.

## 5. Synthesis and crystallization

**Figure 3:**
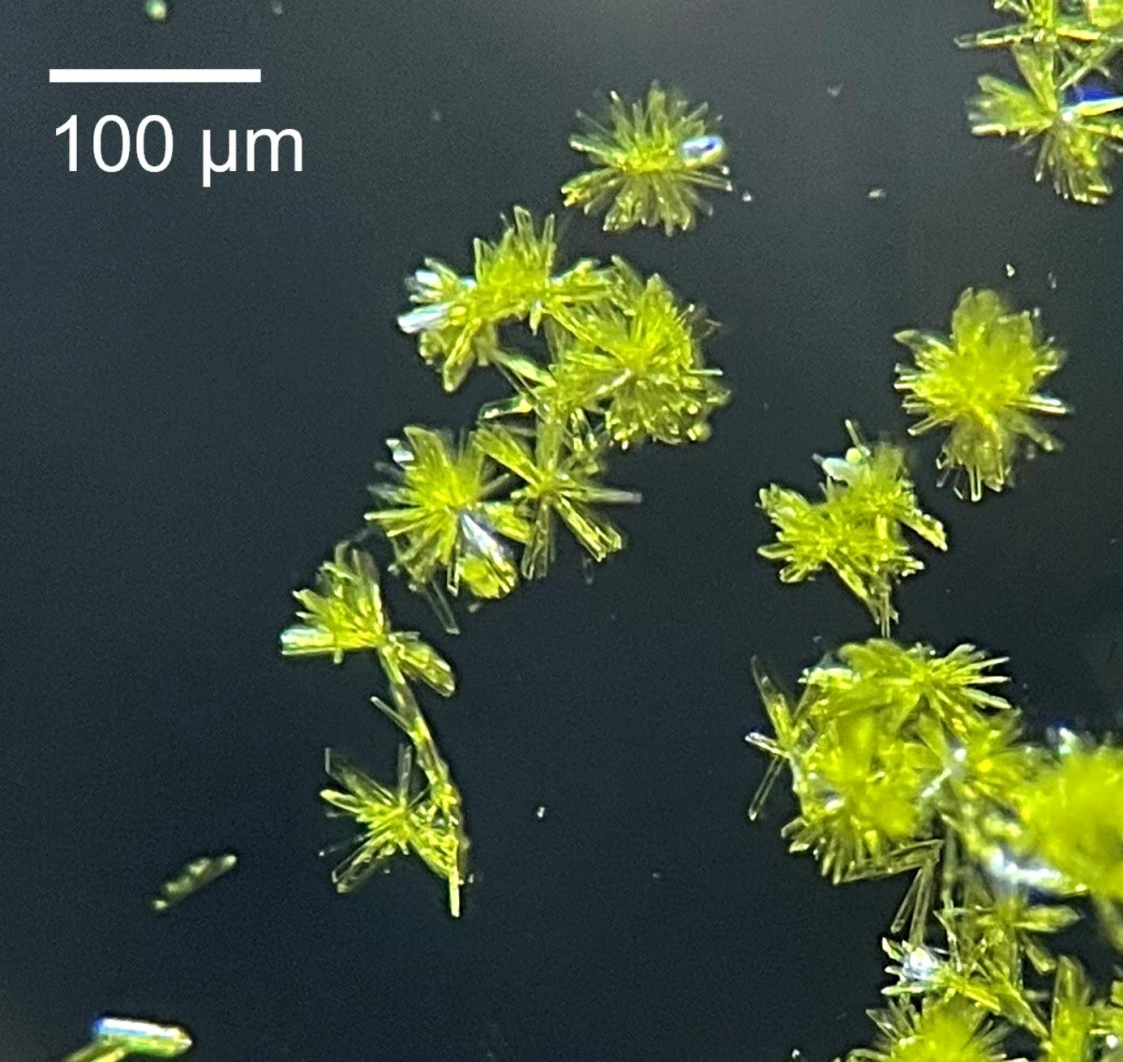
Microscope image of **Sm-1** crystals with scale for reference.

The compound **Sm-1** was synthesized by dissolving 10 mg of Sm^III^ chloride hexa­hydrate (SmCl_3_·6H_2_O, Strem Chemicals, 99.9%) in 208.5 µL of hydro­chloric acid (HCl, Sigma Aldrich, 37% *w*/*w*). The mixture was slowly heated to dryness, and the residue was dissolved in 500 µL of hydro­bromic acid (HBr, Aldrich, 48% *w*/*w* ACS reagent). The solution was gently heated to dryness and once cooled, the residue was dissolved in 655 µL ethanol (Fisher, 200 proof) to form a 0.042 *M* Sm^III^ solution with a pH near 1.4 (*Solution A*). A 0.105 *M o*-vanillin solution (*Solution B*) was prepared by dissolving *o*-vanillin (TCI, >99.0%) in an ethanol/aceto­nitrile (1:1, aceto­nitrile: Fisher, 99.5% certified ACS) mixture. The following were added to a 4 mL glass reaction vial: 100 µL *Solution A*, 400 µL *Solution B*, and 33.4 µL 0.5 *M* NaOH (aqueous, Sigma Aldrich, >98.0%), yielding a yellow solution with a pH of 7.7. The vial was covered with parafilm that had a small slash in it to allow slow evaporation of the solvent. After 4 days, yellow acicular crystals grew from the reaction solution in radial bursts (Fig. 3 ▸). The synthesis of **Sm-1** has an 80% yield. Several synthetic variations were explored to improve the single-crystal diffraction quality. Adding an additional equivalent of NaOH brought the initial pH to ∼8.5 and yielded the same phase, but the crystals were too small for single-crystal studies. Decreasing the NaOH equivalents (in the pH range of 2–4) did not yield any quality crystalline product upon evaporation. In addition, simply starting with SmCl_3_·6H_2_O salt, instead of the HCl/HBr Sm stock protocol, indeed crystallized **Sm-1**; however, these were also too small for individual manipulation. Although not reported here, the synthesis was developed as an analogue for transuranic chemistry, in which strong acid stock solutions are a practicality and serve as redox control.

## 6. Experimental details

**Sm-1** crystals were harvested, washed with ethanol, and mounted to MiTeGen MicroMounts from immersion oil. Data were collected on a Bruker D8 Venture diffractometer equipped with a Photon III detector using a Mo anode micro-focus source (diamond IμS 3.0) and φ and ω scans, at 100 K. The collection strategy was calculated factoring in the known symmetry and collected with at least triplicate multiplicity. The data were reduced using *SAINT* (Bruker, 2014 ▸) and multi-scan absorption correction was applied using *SADABS* (Krause *et al.*, 2015 ▸), both within the *APEX4* software (Bruker, 2014 ▸). Using *Olex2* (Dolomanov *et al.*, 2009 ▸), the structure was solved with the *SHELXT* (Sheldrick, 2015*a* ▸) structure solution program and refined with the *SHELXL* (Sheldrick, 2015*b* ▸) refinement package using least-squares minimization. Additional experimental and instrumentation details on powder X-ray diffraction, infrared spectroscopy, and diffuse reflectance spectroscopy can be found in the supporting information.

## 7. Refinement

Crystal data, data collection, and structure refinement details of **Sm-1** are summarized in Table 2 ▸. The H atoms associated with the carbon atoms were affixed to the respective parent atoms using a riding model. Residual electron density of disordered solvent mol­ecules in the void space could not be reasonably modeled, thus the SQUEEZE function was applied *via PLATON* (Spek, 2015 ▸, 2020 ▸). A total of 47 electrons were accounted for by SQUEEZE and removed. This amounts to about 2 solvent mol­ecules (aceto­nitrile and/or ethanol) per unit cell. While most of the reaction medium was aceto­nitrile and ethanol, water mol­ecules are also possible from the aqueous NaOH spike. The **Sm-1** single crystals diffracted weakly, perhaps owing to the small crystal size. Attempts to crystallize and select higher quality single crystals were unsuccessful. Bond-valence analysis on the metal centers yields summations of 3.30 and 0.98 for Sm^III^ and Na^I^, respectively (Brown & Altermatt, 1985 ▸; Yee *et al.*, 2019 ▸).

## Supplementary Material

Crystal structure: contains datablock(s) I. DOI: 10.1107/S2056989024001051/oo2002sup1.cif

Structure factors: contains datablock(s) I. DOI: 10.1107/S2056989024001051/oo2002Isup2.hkl

Supporting information file. DOI: 10.1107/S2056989024001051/oo2002Isup4.mol

Word document (Characterization details, experimental diffractogram of Sm-1, and FTIR spectrum and diffuse reflectance spectrum of Sm-1 are provided.). DOI: 10.1107/S2056989024001051/oo2002sup3.docx

CCDC reference: 2329845

Additional supporting information:  crystallographic information; 3D view; checkCIF report

## Figures and Tables

**Table 1:** Atom pairs and distances (Å)

Atom pair	Distance
C11—H11⋯C4	2.716
C16—H16*B*⋯C12	2.851
C16—H16*B*⋯C13	2.888

**Table 2:** Experimental details

Crystal data	
Chemical formula	[SmNa(C_8_H_7_O_3_)_4_][+solvent]
*M*_r_	777.88
Crystal system, space group	Monoclinic, *P*2_1_/*c*
Temperature (K)	100
*a*, *b*, *c* (Å)	11.5512 (7), 24.4768 (14), 12.8355 (6)
β (°)	115.742 (2)
*V* (Å^3^)	3268.9 (3)
*Z*	4
Radiation type	Mo *K*α
μ (mm^−1^)	1.87
Crystal size (mm)	0.05 × 0.01 × 0.002

Data collection	
Diffractometer	Bruker D8 Venture with photon detector
Absorption correction	Multi-scan (*SADABS*; Krause *et al.*, 2015 ▸)
No. of measured, independent and observed [*I* > 2σ(*I*)] reflections	41476, 6204, 4562
*R*_int_	0.147
(sin θ/λ)_max_ (Å^−1^)	0.610

Refinement	
*R*[*F* ^2^ > 2σ(*F* ^2^)], *wR*(*F* ^2^), *S*	0.050, 0.108, 1.02
No. of reflections	6204
No. of parameters	419
H-atom treatment	H-atom parameters constrained
Δρ_max_, Δρ_min_ (e Å^−3^)	0.76, −1.13

Computer programs: *APEX4* and *SAINT* (Bruker, 2014 ▸), *SHELXT2018/2* (Sheldrick, 2015*a* ▸), *SHELXL2018/3* (Sheldrick, 2015*b* ▸), and *OLEX2* (Dolomanov *et al.*, 2009 ▸).

## supplementary crystallographic information

### Crystal data

**Table:**

[SmNa(C_8_H_7_O_3_)_4_][+solvent]	*F*(000) = 1556
*M**_r_* = 777.88	*D*_x_ = 1.581 Mg m^−^^3^
Monoclinic, *P*2_1_/*c*	Mo *K*α radiation, λ = 0.71073 Å
*a* = 11.5512 (7) Å	Cell parameters from 6204 reflections
*b* = 24.4768 (14) Å	θ = 2.4–25.1°
*c* = 12.8355 (6) Å	µ = 1.87 mm^−^^1^
β = 115.742 (2)°	*T* = 100 K
*V* = 3268.9 (3) Å^3^	Needle, yellow
*Z* = 4	0.05 × 0.01 × 0.002 mm

### Data collection

**Table:**

Bruker D8 Venture with photon detector diffractometer	4562 reflections with *I* > 2σ(*I*)
Radiation source: Microfocus sealed source	*R*_int_ = 0.147
φ and ω scans	θ_max_ = 25.7°, θ_min_ = 2.0°
Absorption correction: multi-scan (SADABS; Krause *et al.*, 2015)	*h* = −14→14
	*k* = −29→29
41476 measured reflections	*l* = −15→15
6204 independent reflections	

### Refinement

**Table:**

Refinement on *F*^2^	Primary atom site location: dual
Least-squares matrix: full	Hydrogen site location: inferred from neighbouring sites
*R*[*F*^2^ > 2σ(*F*^2^)] = 0.050	H-atom parameters constrained
*wR*(*F*^2^) = 0.108	*w* = 1/[σ^2^(*F*_o_^2^) + (0.0186*P*)^2^ + 17.6315*P*] where *P* = (*F*_o_^2^ + 2*F*_c_^2^)/3
*S* = 1.02	(Δ/σ)_max_ = 0.002
6204 reflections	Δρ_max_ = 0.76 e Å^−^^3^
419 parameters	Δρ_min_ = −1.13 e Å^−^^3^
0 restraints	

### Special details

**Table:**

Geometry. All esds (except the esd in the dihedral angle between two l.s. planes) are estimated using the full covariance matrix. The cell esds are taken into account individually in the estimation of esds in distances, angles and torsion angles; correlations between esds in cell parameters are only used when they are defined by crystal symmetry. An approximate (isotropic) treatment of cell esds is used for estimating esds involving l.s. planes.

### Fractional atomic coordinates and isotropic or equivalent isotropic displacement parameters (Å^2^)

**Table:**

	*x*	*y*	*z*	*U*_iso_*/*U*_eq_	
Sm1	0.54972 (3)	0.69282 (2)	0.62323 (3)	0.01414 (10)	
Na2	0.5507 (2)	0.70628 (10)	0.9140 (2)	0.0195 (6)	
O11	0.3989 (4)	0.75247 (18)	0.4906 (4)	0.0175 (10)	
O9	0.7502 (4)	0.84841 (19)	0.5347 (4)	0.0222 (10)	
O6	0.4809 (4)	0.61822 (18)	0.9430 (4)	0.0194 (10)	
O3	0.8796 (5)	0.55301 (19)	0.7600 (4)	0.0270 (11)	
O8	0.6755 (4)	0.76769 (19)	0.6213 (4)	0.0219 (10)	
O12	0.2499 (4)	0.79508 (19)	0.2874 (4)	0.0252 (11)	
O5	0.4883 (4)	0.64323 (18)	0.7484 (4)	0.0202 (10)	
O2	0.6974 (4)	0.62447 (19)	0.6429 (4)	0.0223 (11)	
O10	0.4681 (4)	0.75359 (18)	0.7262 (4)	0.0203 (10)	
O7	0.7195 (5)	0.70785 (18)	0.8182 (4)	0.0246 (11)	
O1	0.5810 (4)	0.6879 (2)	0.4454 (4)	0.0249 (11)	
O4	0.3873 (5)	0.62479 (19)	0.5110 (4)	0.0252 (11)	
C19	1.0010 (6)	0.7857 (3)	0.8527 (6)	0.0229 (15)	
H19	1.056559	0.770846	0.925847	0.027*	
C4	0.9473 (6)	0.5771 (3)	0.5065 (6)	0.0242 (15)	
H4	1.006658	0.566356	0.477312	0.029*	
C22	0.8385 (6)	0.8297 (3)	0.6396 (6)	0.0192 (14)	
C15	0.4218 (6)	0.6003 (2)	0.7474 (6)	0.0145 (13)	
C6	0.8746 (6)	0.5710 (3)	0.6568 (6)	0.0199 (14)	
C21	0.9621 (6)	0.8489 (3)	0.6987 (6)	0.0211 (15)	
H21	0.991966	0.877738	0.667205	0.025*	
C7	0.7722 (6)	0.6096 (3)	0.5976 (6)	0.0160 (13)	
C23	0.7901 (6)	0.7854 (3)	0.6825 (6)	0.0180 (14)	
C18	0.8738 (6)	0.7657 (3)	0.7937 (6)	0.0195 (14)	
C30	0.2253 (6)	0.8102 (3)	0.3781 (5)	0.0196 (13)	
C25	0.3742 (7)	0.7852 (3)	0.6983 (6)	0.0214 (15)	
H25	0.357064	0.799463	0.758981	0.026*	
C31	0.3105 (6)	0.7860 (3)	0.4853 (6)	0.0185 (14)	
C17	0.8289 (7)	0.7282 (3)	0.8549 (6)	0.0203 (15)	
H17	0.888831	0.717983	0.930675	0.024*	
C20	1.0441 (7)	0.8258 (3)	0.8057 (6)	0.0268 (16)	
H20	1.130164	0.838390	0.845323	0.032*	
C29	0.1314 (7)	0.8462 (3)	0.3702 (7)	0.0271 (16)	
H29	0.077035	0.861599	0.297210	0.033*	
C3	0.8513 (6)	0.6128 (3)	0.4460 (6)	0.0215 (15)	
H3	0.843275	0.626777	0.374140	0.026*	
C26	0.2898 (6)	0.8020 (3)	0.5821 (6)	0.0196 (14)	
C14	0.4154 (6)	0.5839 (3)	0.8518 (6)	0.0185 (14)	
C5	0.9586 (6)	0.5560 (3)	0.6128 (6)	0.0216 (15)	
H5	1.025602	0.530931	0.654560	0.026*	
C28	0.1143 (7)	0.8608 (3)	0.4684 (7)	0.0330 (19)	
H28	0.047552	0.885288	0.461531	0.040*	
C9	0.3416 (7)	0.5839 (3)	0.5370 (6)	0.0272 (16)	
H9	0.294351	0.559717	0.475132	0.033*	
C1	0.6639 (7)	0.6662 (3)	0.4223 (6)	0.0211 (15)	
H1	0.660792	0.675538	0.349272	0.025*	
C2	0.7627 (6)	0.6294 (3)	0.4899 (6)	0.0183 (14)	
C11	0.2853 (7)	0.5207 (3)	0.6534 (6)	0.0255 (16)	
H11	0.240991	0.499080	0.586178	0.031*	
C10	0.3508 (7)	0.5683 (3)	0.6477 (6)	0.0218 (15)	
C16	0.4904 (7)	0.6026 (3)	1.0538 (6)	0.0238 (16)	
H16A	0.542757	0.629416	1.111908	0.036*	
H16B	0.530618	0.566523	1.074545	0.036*	
H16C	0.404221	0.601242	1.050900	0.036*	
C12	0.2858 (7)	0.5058 (3)	0.7564 (7)	0.0280 (17)	
H12	0.242187	0.473566	0.760530	0.034*	
C32	0.1628 (7)	0.8160 (3)	0.1762 (6)	0.0316 (18)	
H32A	0.190174	0.803721	0.117616	0.047*	
H32B	0.163046	0.856006	0.178571	0.047*	
H32C	0.075922	0.802486	0.156396	0.047*	
C13	0.3503 (7)	0.5377 (3)	0.8560 (6)	0.0227 (15)	
H13	0.348877	0.527208	0.926644	0.027*	
C27	0.1937 (7)	0.8398 (3)	0.5735 (7)	0.0319 (18)	
H27	0.184498	0.850406	0.640731	0.038*	
C8	0.9725 (8)	0.5111 (3)	0.8178 (7)	0.039 (2)	
H8A	0.960792	0.480832	0.764274	0.059*	
H8B	0.960716	0.497837	0.884633	0.059*	
H8C	1.059277	0.526134	0.844102	0.059*	
C24	0.7805 (7)	0.8960 (3)	0.4863 (7)	0.0286 (17)	
H24A	0.857600	0.888943	0.474678	0.043*	
H24B	0.796381	0.926870	0.539359	0.043*	
H24C	0.708379	0.904619	0.411926	0.043*	

### Atomic displacement parameters (Å^2^)

**Table:**

	*U*^11^	*U*^22^	*U*^33^	*U*^12^	*U*^13^	*U*^23^
Sm1	0.01358 (16)	0.01627 (16)	0.01311 (16)	0.00116 (16)	0.00628 (12)	0.00091 (16)
Na2	0.0222 (14)	0.0203 (14)	0.0167 (13)	−0.0018 (10)	0.0090 (11)	−0.0019 (10)
O11	0.012 (2)	0.019 (2)	0.018 (2)	0.0034 (18)	0.0026 (19)	0.0009 (19)
O9	0.021 (3)	0.025 (3)	0.021 (3)	−0.002 (2)	0.009 (2)	0.008 (2)
O6	0.023 (3)	0.021 (2)	0.015 (2)	−0.003 (2)	0.009 (2)	0.0001 (19)
O3	0.026 (3)	0.026 (3)	0.025 (3)	0.009 (2)	0.008 (2)	0.010 (2)
O8	0.015 (2)	0.031 (3)	0.014 (2)	−0.006 (2)	0.001 (2)	0.002 (2)
O12	0.021 (3)	0.030 (3)	0.020 (3)	0.006 (2)	0.005 (2)	0.005 (2)
O5	0.021 (3)	0.021 (2)	0.022 (3)	−0.001 (2)	0.011 (2)	−0.004 (2)
O2	0.021 (3)	0.028 (3)	0.020 (3)	0.014 (2)	0.010 (2)	0.010 (2)
O10	0.025 (3)	0.018 (2)	0.018 (2)	0.000 (2)	0.010 (2)	−0.0058 (19)
O7	0.027 (3)	0.024 (3)	0.021 (3)	−0.007 (2)	0.009 (2)	0.003 (2)
O1	0.026 (3)	0.033 (3)	0.021 (2)	0.011 (2)	0.014 (2)	0.005 (2)
O4	0.029 (3)	0.029 (3)	0.018 (3)	−0.010 (2)	0.011 (2)	−0.004 (2)
C19	0.015 (4)	0.032 (4)	0.017 (4)	0.007 (3)	0.003 (3)	−0.002 (3)
C4	0.016 (4)	0.027 (4)	0.028 (4)	−0.002 (3)	0.009 (3)	−0.004 (3)
C22	0.019 (4)	0.023 (3)	0.017 (3)	0.004 (3)	0.009 (3)	0.002 (3)
C15	0.005 (3)	0.015 (3)	0.022 (4)	0.003 (2)	0.004 (3)	0.003 (3)
C6	0.020 (4)	0.014 (3)	0.025 (4)	0.002 (3)	0.009 (3)	0.003 (3)
C21	0.016 (4)	0.021 (3)	0.029 (4)	−0.004 (3)	0.012 (3)	−0.006 (3)
C7	0.011 (3)	0.017 (3)	0.020 (3)	0.000 (3)	0.006 (3)	−0.004 (3)
C23	0.013 (3)	0.020 (3)	0.027 (4)	0.005 (3)	0.014 (3)	0.000 (3)
C18	0.013 (3)	0.028 (4)	0.018 (3)	0.002 (3)	0.007 (3)	−0.002 (3)
C30	0.012 (3)	0.021 (3)	0.022 (3)	−0.001 (3)	0.004 (3)	0.005 (3)
C25	0.026 (4)	0.021 (3)	0.023 (4)	−0.006 (3)	0.015 (3)	−0.004 (3)
C31	0.008 (3)	0.018 (3)	0.031 (4)	−0.002 (3)	0.010 (3)	0.000 (3)
C17	0.023 (4)	0.020 (3)	0.016 (3)	0.007 (3)	0.007 (3)	0.001 (3)
C20	0.019 (4)	0.034 (4)	0.023 (4)	−0.007 (3)	0.005 (3)	−0.010 (3)
C29	0.016 (4)	0.031 (4)	0.034 (4)	0.007 (3)	0.011 (3)	0.011 (3)
C3	0.021 (4)	0.019 (3)	0.029 (4)	−0.004 (3)	0.014 (3)	−0.005 (3)
C26	0.013 (3)	0.021 (3)	0.022 (3)	0.002 (3)	0.005 (3)	0.005 (3)
C14	0.014 (3)	0.018 (3)	0.025 (4)	0.002 (3)	0.009 (3)	0.005 (3)
C5	0.013 (3)	0.017 (3)	0.030 (4)	0.003 (3)	0.005 (3)	−0.001 (3)
C28	0.021 (4)	0.037 (4)	0.050 (5)	0.008 (3)	0.024 (4)	0.010 (4)
C9	0.028 (4)	0.023 (4)	0.034 (4)	−0.006 (3)	0.016 (4)	−0.013 (3)
C1	0.029 (4)	0.015 (3)	0.021 (4)	0.002 (3)	0.013 (3)	0.004 (3)
C2	0.018 (3)	0.018 (3)	0.022 (4)	0.000 (3)	0.011 (3)	−0.001 (3)
C11	0.022 (4)	0.019 (4)	0.029 (4)	−0.004 (3)	0.005 (3)	−0.005 (3)
C10	0.021 (4)	0.018 (3)	0.024 (4)	0.000 (3)	0.008 (3)	−0.002 (3)
C16	0.039 (4)	0.022 (4)	0.013 (3)	0.002 (3)	0.015 (3)	0.009 (3)
C12	0.022 (4)	0.018 (4)	0.046 (5)	−0.007 (3)	0.016 (4)	−0.002 (3)
C32	0.026 (4)	0.041 (5)	0.016 (4)	0.007 (3)	−0.002 (3)	0.005 (3)
C13	0.026 (4)	0.018 (3)	0.029 (4)	0.000 (3)	0.017 (3)	0.004 (3)
C27	0.029 (4)	0.034 (4)	0.042 (5)	0.007 (3)	0.024 (4)	0.000 (4)
C8	0.036 (5)	0.041 (5)	0.037 (5)	0.016 (4)	0.013 (4)	0.011 (4)
C24	0.024 (4)	0.026 (4)	0.037 (4)	0.001 (3)	0.015 (4)	0.005 (3)

### Geometric parameters (Å, º)

**Table:**

Sm1—Na2	3.742 (2)	C6—C5	1.368 (9)
Sm1—Na2^i^	3.652 (2)	C21—H21	0.9500
Sm1—O11	2.343 (4)	C21—C20	1.404 (10)
Sm1—O8	2.346 (4)	C7—C2	1.424 (9)
Sm1—O5	2.355 (4)	C23—C18	1.417 (9)
Sm1—O2	2.323 (4)	C18—C17	1.444 (9)
Sm1—O10	2.435 (4)	C30—C31	1.427 (9)
Sm1—O7	2.446 (5)	C30—C29	1.366 (9)
Sm1—O1	2.464 (4)	C25—H25	0.9500
Sm1—O4	2.454 (5)	C25—C26	1.442 (9)
Na2—O11^ii^	2.561 (5)	C31—C26	1.419 (9)
Na2—O9^ii^	2.530 (5)	C17—H17	0.9500
Na2—O6	2.386 (5)	C20—H20	0.9500
Na2—O8^ii^	2.496 (5)	C29—H29	0.9500
Na2—O5	2.468 (5)	C29—C28	1.405 (10)
Na2—O10	2.463 (5)	C3—H3	0.9500
Na2—O7	2.720 (5)	C3—C2	1.424 (9)
Na2—O1^ii^	2.622 (5)	C26—C27	1.412 (9)
O11—C31	1.288 (7)	C14—C13	1.371 (9)
O9—C22	1.367 (8)	C5—H5	0.9500
O9—C24	1.433 (8)	C28—H28	0.9500
O6—C14	1.372 (8)	C28—C27	1.361 (11)
O6—C16	1.430 (7)	C9—H9	0.9500
O3—C6	1.373 (8)	C9—C10	1.429 (10)
O3—C8	1.436 (8)	C1—H1	0.9500
O8—C23	1.286 (8)	C1—C2	1.418 (9)
O12—C30	1.365 (8)	C11—H11	0.9500
O12—C32	1.437 (8)	C11—C10	1.409 (9)
O5—C15	1.300 (7)	C11—C12	1.369 (10)
O2—C7	1.286 (7)	C16—H16A	0.9800
O10—C25	1.251 (8)	C16—H16B	0.9800
O7—C17	1.245 (8)	C16—H16C	0.9800
O1—C1	1.239 (8)	C12—H12	0.9500
O4—C9	1.243 (8)	C12—C13	1.404 (10)
C19—H19	0.9500	C32—H32A	0.9800
C19—C18	1.415 (9)	C32—H32B	0.9800
C19—C20	1.356 (10)	C32—H32C	0.9800
C4—H4	0.9500	C13—H13	0.9500
C4—C3	1.360 (10)	C27—H27	0.9500
C4—C5	1.411 (10)	C8—H8A	0.9800
C22—C21	1.376 (9)	C8—H8B	0.9800
C22—C23	1.434 (9)	C8—H8C	0.9800
C15—C14	1.431 (9)	C24—H24A	0.9800
C15—C10	1.418 (9)	C24—H24B	0.9800
C6—C7	1.444 (9)	C24—H24C	0.9800
			
Na2^i^—Sm1—Na2	132.39 (3)	C17—O7—Na2	130.1 (4)
O11—Sm1—Na2	110.43 (11)	Sm1—O1—Na2^i^	91.74 (16)
O11—Sm1—Na2^i^	44.20 (11)	C1—O1—Sm1	133.1 (4)
O11—Sm1—O8	76.94 (16)	C1—O1—Na2^i^	116.9 (4)
O11—Sm1—O5	117.89 (15)	C9—O4—Sm1	133.9 (5)
O11—Sm1—O10	70.90 (15)	C18—C19—H19	119.7
O11—Sm1—O7	131.50 (15)	C20—C19—H19	119.7
O11—Sm1—O1	73.70 (15)	C20—C19—C18	120.6 (7)
O11—Sm1—O4	81.87 (16)	C3—C4—H4	120.1
O8—Sm1—Na2^i^	42.62 (12)	C3—C4—C5	119.8 (6)
O8—Sm1—Na2	102.02 (12)	C5—C4—H4	120.1
O8—Sm1—O5	141.38 (16)	O9—C22—C21	125.5 (6)
O8—Sm1—O10	85.20 (15)	O9—C22—C23	112.5 (6)
O8—Sm1—O7	70.62 (15)	C21—C22—C23	122.0 (6)
O8—Sm1—O1	71.81 (16)	O5—C15—C14	119.3 (6)
O8—Sm1—O4	147.48 (15)	O5—C15—C10	124.2 (6)
O5—Sm1—Na2^i^	161.47 (12)	C10—C15—C14	116.5 (6)
O5—Sm1—Na2	40.22 (11)	O3—C6—C7	113.5 (5)
O5—Sm1—O10	69.04 (15)	C5—C6—O3	124.8 (6)
O5—Sm1—O7	74.01 (15)	C5—C6—C7	121.6 (6)
O5—Sm1—O1	144.62 (16)	C22—C21—H21	120.0
O5—Sm1—O4	70.78 (15)	C22—C21—C20	120.0 (6)
O2—Sm1—Na2^i^	109.28 (11)	C20—C21—H21	120.0
O2—Sm1—Na2	105.73 (11)	O2—C7—C6	120.3 (6)
O2—Sm1—O11	143.78 (15)	O2—C7—C2	124.0 (6)
O2—Sm1—O8	97.74 (16)	C2—C7—C6	115.7 (5)
O2—Sm1—O5	88.81 (15)	O8—C23—C22	119.4 (6)
O2—Sm1—O10	145.08 (16)	O8—C23—C18	124.9 (6)
O2—Sm1—O7	76.86 (16)	C18—C23—C22	115.7 (6)
O2—Sm1—O1	70.67 (15)	C19—C18—C23	121.2 (6)
O2—Sm1—O4	85.02 (17)	C19—C18—C17	117.6 (6)
O10—Sm1—Na2^i^	96.41 (11)	C23—C18—C17	121.0 (6)
O10—Sm1—Na2	40.45 (11)	O12—C30—C31	113.4 (6)
O10—Sm1—O7	71.35 (16)	O12—C30—C29	124.3 (6)
O10—Sm1—O1	141.24 (15)	C29—C30—C31	122.3 (6)
O10—Sm1—O4	110.82 (16)	O10—C25—H25	117.1
O7—Sm1—Na2	46.55 (12)	O10—C25—C26	125.8 (6)
O7—Sm1—Na2^i^	113.18 (11)	C26—C25—H25	117.1
O7—Sm1—O1	125.48 (16)	O11—C31—C30	121.0 (6)
O7—Sm1—O4	140.51 (15)	O11—C31—C26	124.1 (6)
O1—Sm1—Na2^i^	45.86 (12)	C26—C31—C30	114.9 (6)
O1—Sm1—Na2	171.95 (12)	O7—C17—C18	126.5 (6)
O4—Sm1—Na2	108.40 (11)	O7—C17—H17	116.8
O4—Sm1—Na2^i^	105.84 (12)	C18—C17—H17	116.8
O4—Sm1—O1	78.76 (16)	C19—C20—C21	120.3 (7)
Sm1^ii^—Na2—Sm1	142.49 (7)	C19—C20—H20	119.9
O11^ii^—Na2—Sm1	135.37 (13)	C21—C20—H20	119.9
O11^ii^—Na2—Sm1^ii^	39.63 (10)	C30—C29—H29	119.6
O11^ii^—Na2—O7	155.90 (17)	C30—C29—C28	120.9 (7)
O11^ii^—Na2—O1^ii^	67.62 (14)	C28—C29—H29	119.6
O9^ii^—Na2—Sm1	99.88 (12)	C4—C3—H3	119.8
O9^ii^—Na2—Sm1^ii^	101.57 (13)	C4—C3—C2	120.3 (7)
O9^ii^—Na2—O11^ii^	124.54 (17)	C2—C3—H3	119.8
O9^ii^—Na2—O7	69.09 (15)	C31—C26—C25	122.1 (6)
O9^ii^—Na2—O1^ii^	113.80 (18)	C27—C26—C25	115.0 (6)
O6—Na2—Sm1	102.78 (13)	C27—C26—C31	122.6 (6)
O6—Na2—Sm1^ii^	112.76 (13)	O6—C14—C15	113.2 (5)
O6—Na2—O11^ii^	87.83 (16)	C13—C14—O6	125.5 (6)
O6—Na2—O9^ii^	72.92 (17)	C13—C14—C15	121.3 (6)
O6—Na2—O8^ii^	98.17 (17)	C4—C5—H5	119.4
O6—Na2—O5	65.02 (16)	C6—C5—C4	121.1 (6)
O6—Na2—O10	124.32 (18)	C6—C5—H5	119.4
O6—Na2—O7	116.13 (17)	C29—C28—H28	120.1
O6—Na2—O1^ii^	154.16 (17)	C27—C28—C29	119.8 (7)
O8^ii^—Na2—Sm1	146.68 (14)	C27—C28—H28	120.1
O8^ii^—Na2—Sm1^ii^	39.52 (11)	O4—C9—H9	115.7
O8^ii^—Na2—O11^ii^	70.44 (15)	O4—C9—C10	128.7 (7)
O8^ii^—Na2—O9^ii^	62.06 (15)	C10—C9—H9	115.7
O8^ii^—Na2—O7	106.41 (17)	O1—C1—H1	115.6
O8^ii^—Na2—O1^ii^	66.87 (16)	O1—C1—C2	128.8 (6)
O5—Na2—Sm1^ii^	164.45 (14)	C2—C1—H1	115.6
O5—Na2—Sm1	38.03 (11)	C7—C2—C3	121.4 (6)
O5—Na2—O11^ii^	125.80 (18)	C1—C2—C7	120.8 (6)
O5—Na2—O9^ii^	92.53 (17)	C1—C2—C3	117.7 (6)
O5—Na2—O8^ii^	153.53 (19)	C10—C11—H11	120.2
O5—Na2—O7	67.52 (15)	C12—C11—H11	120.2
O5—Na2—O1^ii^	136.04 (17)	C12—C11—C10	119.6 (7)
O10—Na2—Sm1	39.91 (10)	C15—C10—C9	121.0 (6)
O10—Na2—Sm1^ii^	106.15 (12)	C11—C10—C15	121.5 (6)
O10—Na2—O11^ii^	98.71 (16)	C11—C10—C9	117.5 (6)
O10—Na2—O9^ii^	135.31 (18)	O6—C16—H16A	109.5
O10—Na2—O8^ii^	136.24 (18)	O6—C16—H16B	109.5
O10—Na2—O5	66.83 (15)	O6—C16—H16C	109.5
O10—Na2—O7	66.42 (16)	H16A—C16—H16B	109.5
O10—Na2—O1^ii^	69.82 (15)	H16A—C16—H16C	109.5
O7—Na2—Sm1^ii^	123.68 (13)	H16B—C16—H16C	109.5
O7—Na2—Sm1	40.76 (11)	C11—C12—H12	119.7
O1^ii^—Na2—Sm1	100.53 (11)	C11—C12—C13	120.6 (6)
O1^ii^—Na2—Sm1^ii^	42.40 (10)	C13—C12—H12	119.7
O1^ii^—Na2—O7	88.94 (15)	O12—C32—H32A	109.5
Sm1—O11—Na2^i^	96.18 (16)	O12—C32—H32B	109.5
C31—O11—Sm1	139.3 (4)	O12—C32—H32C	109.5
C31—O11—Na2^i^	112.8 (4)	H32A—C32—H32B	109.5
C22—O9—Na2^i^	121.2 (4)	H32A—C32—H32C	109.5
C22—O9—C24	118.7 (5)	H32B—C32—H32C	109.5
C24—O9—Na2^i^	119.6 (4)	C14—C13—C12	120.4 (6)
C14—O6—Na2	121.3 (4)	C14—C13—H13	119.8
C14—O6—C16	117.4 (5)	C12—C13—H13	119.8
C16—O6—Na2	120.7 (4)	C26—C27—H27	120.2
C6—O3—C8	115.9 (5)	C28—C27—C26	119.6 (7)
Sm1—O8—Na2^i^	97.86 (17)	C28—C27—H27	120.2
C23—O8—Sm1	137.3 (4)	O3—C8—H8A	109.5
C23—O8—Na2^i^	122.0 (4)	O3—C8—H8B	109.5
C30—O12—C32	115.9 (5)	O3—C8—H8C	109.5
Sm1—O5—Na2	101.75 (17)	H8A—C8—H8B	109.5
C15—O5—Sm1	139.7 (4)	H8A—C8—H8C	109.5
C15—O5—Na2	117.6 (4)	H8B—C8—H8C	109.5
C7—O2—Sm1	139.6 (4)	O9—C24—H24A	109.5
Sm1—O10—Na2	99.63 (17)	O9—C24—H24B	109.5
C25—O10—Sm1	135.6 (4)	O9—C24—H24C	109.5
C25—O10—Na2	119.4 (4)	H24A—C24—H24B	109.5
Sm1—O7—Na2	92.68 (16)	H24A—C24—H24C	109.5
C17—O7—Sm1	132.7 (4)	H24B—C24—H24C	109.5
			
Sm1—O11—C31—C30	−171.4 (4)	O4—C9—C10—C15	−1.8 (12)
Sm1—O11—C31—C26	9.0 (10)	O4—C9—C10—C11	177.4 (7)
Sm1—O8—C23—C22	169.3 (4)	C19—C18—C17—O7	177.7 (6)
Sm1—O8—C23—C18	−13.0 (10)	C4—C3—C2—C7	0.4 (10)
Sm1—O5—C15—C14	177.5 (4)	C4—C3—C2—C1	−179.3 (6)
Sm1—O5—C15—C10	−4.2 (10)	C22—C21—C20—C19	−1.8 (10)
Sm1—O2—C7—C6	163.9 (5)	C22—C23—C18—C19	−5.4 (9)
Sm1—O2—C7—C2	−15.2 (11)	C22—C23—C18—C17	169.0 (6)
Sm1—O10—C25—C26	−10.9 (10)	C15—C14—C13—C12	−1.3 (10)
Sm1—O7—C17—C18	22.4 (10)	C6—C7—C2—C3	−1.5 (9)
Sm1—O1—C1—C2	9.8 (11)	C6—C7—C2—C1	178.2 (6)
Sm1—O4—C9—C10	14.5 (11)	C21—C22—C23—O8	−177.2 (6)
Na2^i^—O11—C31—C30	56.8 (7)	C21—C22—C23—C18	5.0 (9)
Na2^i^—O11—C31—C26	−122.8 (6)	C7—C6—C5—C4	−0.9 (10)
Na2^i^—O9—C22—C21	163.7 (5)	C23—C22—C21—C20	−1.5 (10)
Na2^i^—O9—C22—C23	−14.4 (7)	C23—C18—C17—O7	3.1 (10)
Na2—O6—C14—C15	14.1 (7)	C18—C19—C20—C21	1.3 (10)
Na2—O6—C14—C13	−166.7 (5)	C30—C31—C26—C25	−174.4 (6)
Na2^i^—O8—C23—C22	13.1 (8)	C30—C31—C26—C27	−1.1 (10)
Na2^i^—O8—C23—C18	−169.2 (5)	C30—C29—C28—C27	1.3 (11)
Na2—O5—C15—C14	−15.7 (7)	C25—C26—C27—C28	175.8 (7)
Na2—O5—C15—C10	162.7 (5)	C31—C30—C29—C28	−0.4 (11)
Na2—O10—C25—C26	−158.9 (5)	C31—C26—C27—C28	2.1 (11)
Na2—O7—C17—C18	−126.9 (6)	C20—C19—C18—C23	2.5 (10)
Na2^i^—O1—C1—C2	131.2 (6)	C20—C19—C18—C17	−172.1 (6)
O11—C31—C26—C25	5.3 (10)	C29—C30—C31—O11	−179.4 (6)
O11—C31—C26—C27	178.6 (6)	C29—C30—C31—C26	0.3 (9)
O9—C22—C21—C20	−179.4 (6)	C29—C28—C27—C26	−2.1 (11)
O9—C22—C23—O8	1.1 (8)	C3—C4—C5—C6	−0.3 (10)
O9—C22—C23—C18	−176.8 (5)	C14—C15—C10—C9	174.6 (6)
O6—C14—C13—C12	179.6 (6)	C14—C15—C10—C11	−4.5 (9)
O3—C6—C7—O2	0.9 (9)	C5—C4—C3—C2	0.5 (10)
O3—C6—C7—C2	−179.9 (6)	C5—C6—C7—O2	−177.5 (6)
O3—C6—C5—C4	−179.1 (6)	C5—C6—C7—C2	1.7 (9)
O8—C23—C18—C19	176.8 (6)	C11—C12—C13—C14	−1.0 (11)
O8—C23—C18—C17	−8.8 (10)	C10—C15—C14—O6	−176.8 (5)
O12—C30—C31—O11	−1.3 (9)	C10—C15—C14—C13	3.9 (9)
O12—C30—C31—C26	178.4 (5)	C10—C11—C12—C13	0.4 (11)
O12—C30—C29—C28	−178.3 (6)	C16—O6—C14—C15	−174.3 (5)
O5—C15—C14—O6	1.7 (8)	C16—O6—C14—C13	4.9 (9)
O5—C15—C14—C13	−177.5 (6)	C12—C11—C10—C15	2.5 (10)
O5—C15—C10—C9	−3.9 (10)	C12—C11—C10—C9	−176.7 (7)
O5—C15—C10—C11	177.0 (6)	C32—O12—C30—C31	176.9 (6)
O2—C7—C2—C3	177.7 (6)	C32—O12—C30—C29	−5.1 (10)
O2—C7—C2—C1	−2.6 (10)	C8—O3—C6—C7	174.9 (6)
O10—C25—C26—C31	−3.8 (11)	C8—O3—C6—C5	−6.8 (10)
O10—C25—C26—C27	−177.6 (7)	C24—O9—C22—C21	−7.8 (9)
O1—C1—C2—C7	4.1 (11)	C24—O9—C22—C23	174.1 (5)
O1—C1—C2—C3	−176.2 (7)		

Symmetry codes: (i) *x*, −*y*+3/2, *z*−1/2; (ii) *x*, −*y*+3/2, *z*+1/2.

## References

[bb1] Albrecht, M. (2001). *Chem. Rev.* **101**, 3457–3498. 10.1021/cr0103672 11840991

[bb2] Andruh, M. (2015). *Dalton Trans.* **44**, 16633–16653. 10.1039/c5dt02661j 26282536

[bb3] Brown, I. D. & Altermatt, D. (1985). *Acta Cryst.* B**41**, 244–247.

[bb4] Bruker (2014). *APEX4* and *SAINT*. Bruker AXS Inc., Madison, Wisconsin, USA.

[bb5] Bünzli, J. G. & Piguet, C. (2002). *Chem. Rev.* **102**, 1897–1928. 10.1021/cr010299j 12059257

[bb6] Chaudhari, A. K., Joarder, B., Rivière, E., Rogez, G. & Ghosh, S. K. (2012). *Inorg. Chem.* **51**, 9159–9161. 10.1021/ic3012876 22909388

[bb7] Cheng, J.-W. & Yang, G.-Y. (2017). *Recent Development in Clusters of Rare Earths and Actinides: Chemistry and Materials*, edited by Z. Zheng, pp. 97–119. Berlin, Heidelberg: Springer Berlin Heidelberg.

[bb8] Costes, J., Dahan, F., Duhayon, C. & Mota, A. J. (2015). *Polyhedron*, **96**, 51–56.

[bb9] Costes, J., Dahan, F., Vendier, L., Shova, S., Lorusso, G. & Evangelisti, M. (2018). *Dalton Trans.* **47**, 1106–1116. 10.1039/c7dt04293k 29265143

[bb10] Dolomanov, O. V., Bourhis, L. J., Gildea, R. J., Howard, J. A. K. & Puschmann, H. (2009). *J. Appl. Cryst.* **42**, 339–341.

[bb11] Griffiths, K., Kumar, P., Mattock, J. D., Abdul-Sada, A., Pitak, M. B., Coles, S. J., Navarro, O., Vargas, A. & Kostakis, G. E. (2016). *Inorg. Chem.* **55**, 6988–6994. 10.1021/acs.inorgchem.6b00720 27355452

[bb12] Groom, C. R., Bruno, I. J., Lightfoot, M. P. & Ward, S. C. (2016). *Acta Cryst.* B**72**, 171–179. 10.1107/S2052520616003954 PMC4822653 27048719

[bb13] Heuer-Jungemann, A., Feliu, N., Bakaimi, I., Hamaly, M., Alkilany, A., Chakraborty, I., Masood, A., Casula, M. F., Kostopoulou, A., Oh, E., Susumu, K., Stewart, M. H., Medintz, I. L., Stratakis, E., Parak, W. J. & Kanaras, A. G. (2019). *Chem. Rev.* **119**, 97–119. 10.1021/acs.chemrev.8b00733 30920815

[bb14] Kırpık, H., Kose, M., Elsegood, M. & Carpenter-Warren, C. L. (2019). *J. Mol. Struct.* **1175**, 882–888.

[bb15] Krause, L., Herbst-Irmer, R., Sheldrick, G. M. & Stalke, D. (2015). *J. Appl. Cryst.* **48**, 3–10. 10.1107/S1600576714022985 PMC4453166 26089746

[bb16] Li, X., Zhao, L., Wu, J., Shi, W., Struch, N., Lutzen, A., Powell, A. K., Cheng, P. & Tang, J. (2022). *Chem. Sci.* **13**, 10048–10056. 10.1039/d2sc03156f PMC9430530 36128245

[bb17] Ma, J., Ma, T., Qian, R., Zhou, L., Guo, Q., Yang, J. & Yang, Q. (2021). *Inorg. Chem.* **60**, 7937–7951. 10.1021/acs.inorgchem.1c00462 34015217

[bb18] Novitchi, G., Pilet, G., Ungur, L., Moshchalkov, V. V., Wernsdorfer, W., Chibotaru, L. F., Luneau, D. & Powell, A. K. (2012). *Chem. Sci.* **3**, 1169–1176.

[bb19] Peng, G., Ma, L., Cai, J., Liang, L., Deng, H. & Kostakis, G. E. (2011). *Cryst. Growth Des.* **11**, 2485–2492.

[bb20] Sheldrick, G. M. (2015*a*). *Acta Cryst.* A**71**, 3–8.

[bb21] Sheldrick, G. M. (2015*b*). *Acta Cryst.* C**71**, 3–8.

[bb22] Song, X., Liu, P., Wang, C., Liu, Y., Liu, W. & Zhang, M. (2017). *RSC Adv.* **7**, 22692–22698.

[bb23] Spek, A. L. (2015). *Acta Cryst.* C**71**, 9–18. 10.1107/S2053229614024929 25567569

[bb24] Spek, A. L. (2020). *Acta Cryst.* E**76**, 1–11. 10.1107/S2056989019016244 PMC6944088 31921444

[bb25] Yee, T. A., Suescun, L. & Rabuffetti, F. A. (2019). *J. Solid State Chem.* **270**, 242–246.

