# Supplementary material for: Structural characterization of a new samarium–sodium heterometallic coordination polymer
Source: Acta Crystallogr E Crystallogr Commun. 2024 Feb 6;80(Pt 3):267–70. doi: 10.1107/S2056989024001051 (PMC10915667; doi:10.1107/S2056989024001051)
Supplement: Supplementary file 4 [file e-80-00267-sup3.docx]

**Structural characterization of a new samarium—sodium heterometallic coordination polymer**

Ashley M. Hastings,^1,2,3†^Ashley Williams,^1†^ Robert G. Surbella III,^1^ Amy E. Hixon,^2^ and Ana Arteaga^1^*

^1^Pacific Northwest National Laboratory, Richland, Washington 99354, USA

^2^Department of Civil and Environmental Engineering and Earth Sciences, University of Notre Dame, Notre Dame, Indiana 46556, USA

^3^Current affiliation: Lawrence Livermore National Laboratory, Livermore, California 94550, USA

^†^These authors contributed equally to this work.

*e-mail: ana.arteaga@pnnl.gov

| **Table of Contents** | **Page #** |
| --- | --- |
| Figure S1……………………………….. | S2 |
| Powder X-ray Diffraction Data………… | S3 |
| Infrared Spectroscopy………………….. | S4 |
| Diffuse Reflectance Spectroscopy……... | S5 |
| References…………………………….... | S5 |

**
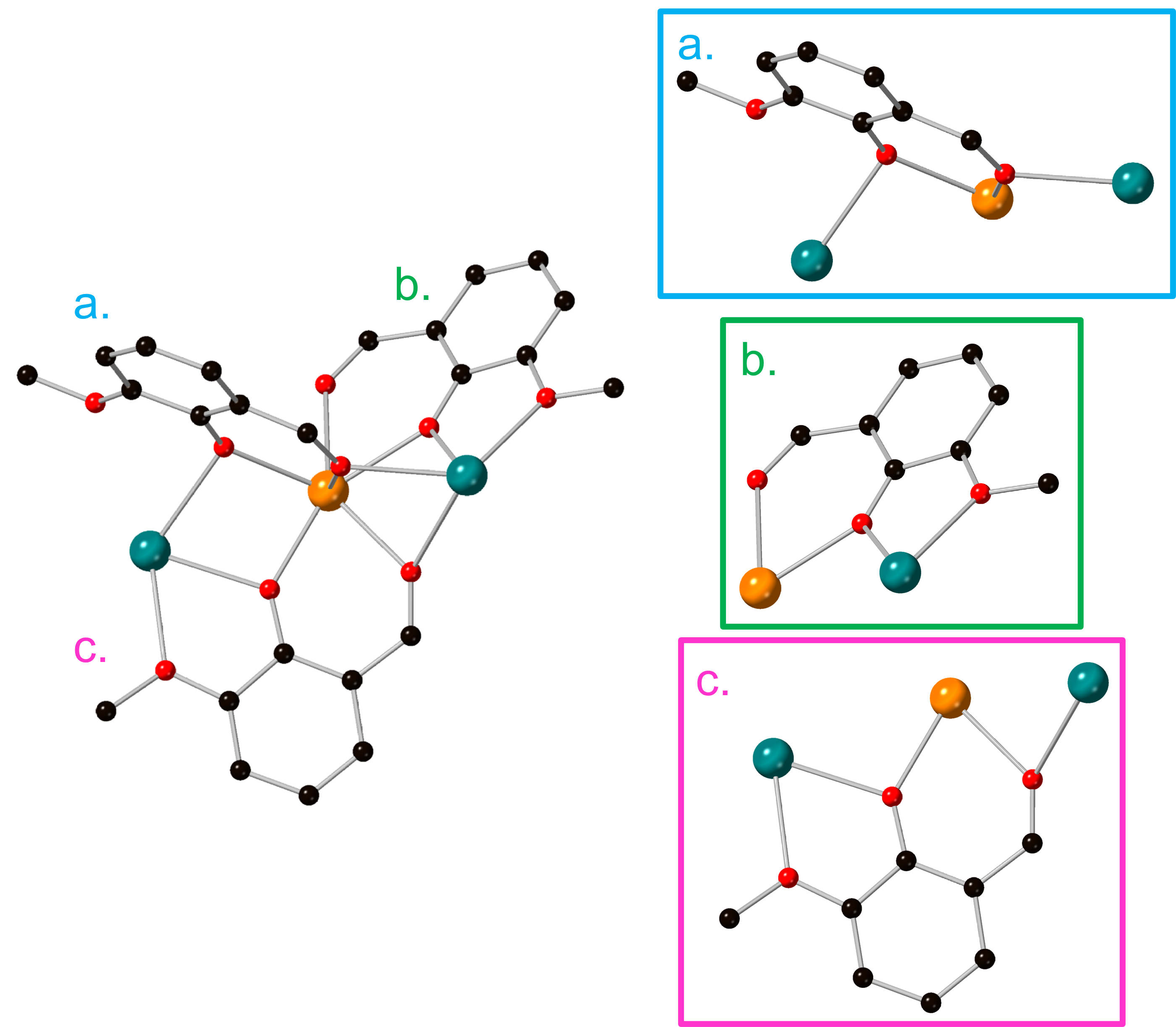
**

**Figure S1.** (left) Ball and stick representation of **Sm-1** showing the Sm(III) and Na(I) atoms alternate and are bridged together by three µ_2_-*o*-vanillin ligands, and (right) individual representation of each *o*-vanillin ligand displaying the unique bonding environments through the phenoxo, aldehydic, and methoxy group. The Sm, Na, C, and O atoms are depicted as orange, teal, black, and red ellipsoids, respectively.

**Powder X-ray Diffraction**

Powder X-ray diffraction was used to compare the experimental and calculated diffractograms of **Sm-1**, Figure S2. The sample was prepared by an ethanolic dropcast of **Sm-1** onto a zero-background plate that was loaded onto a Bruker manufactured sample holder (item# C79298A3244D97). Data were collected using a Rigaku Ultima IV diffractometer outfitted with Cu sealed tube X-ray tube, with a scan rate of 1° min^-1^ and 0.02° step size from 5–50° 2θ. Divergence and incident slits of 5 mm and 0.5 mm were used with a Ni foil filter to reduce the Kβ contributions from the Cu source. Some preferred orientation is evident, but no additional crystalline phases are observed.


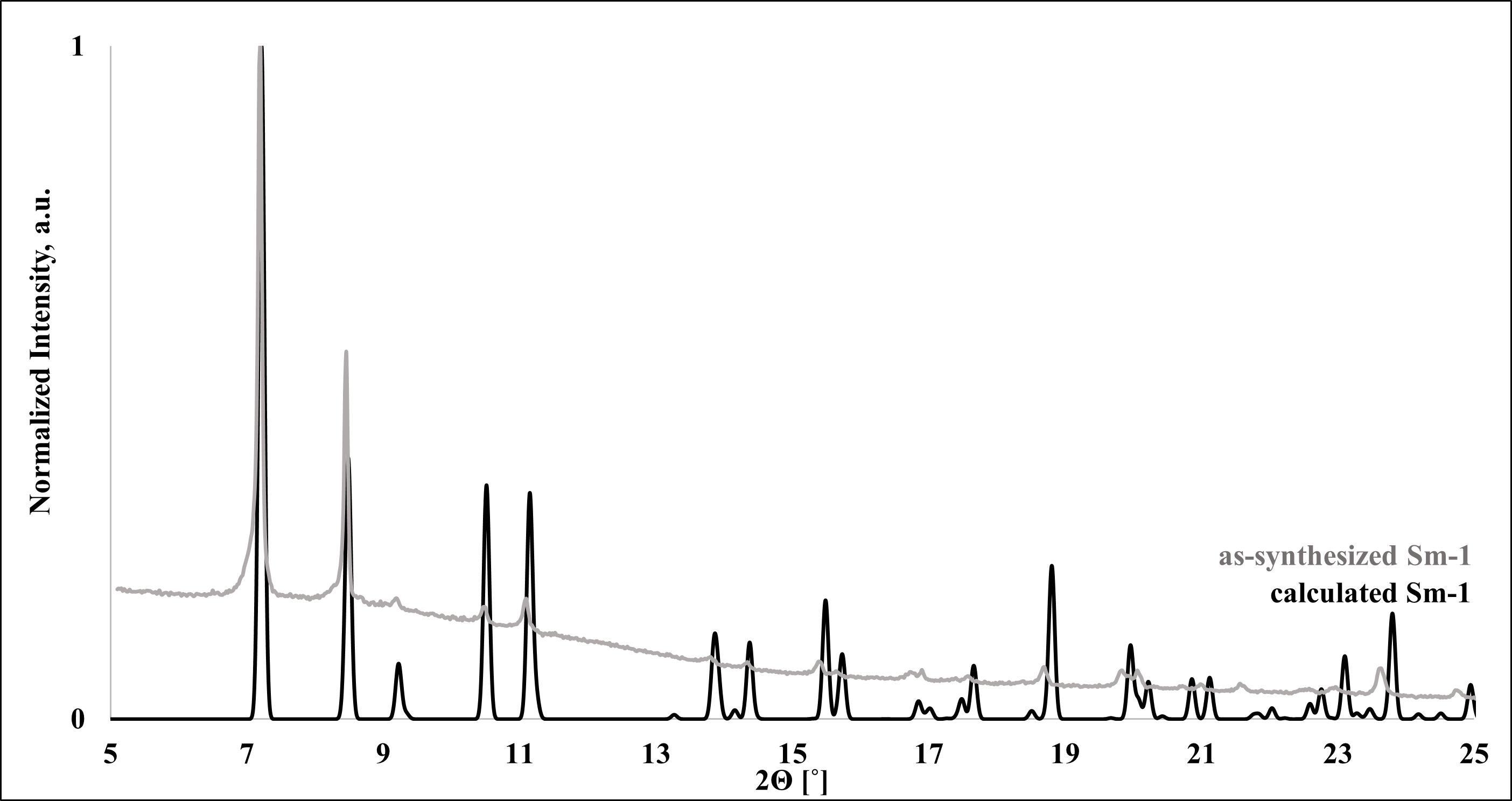


**Figure S2.** The experimental (gray) and calculated (black) diffractograms of **Sm-1.** The calculated pattern was obtained from the single crystal X-ray diffraction data.

**Attenuated Total Reflectance Fourier Transform Infrared spectroscopy (ATR-FTIR)** was performed on **Sm-1**. The data were collected using a Bruker Lumos – FTIR spectrometer outfitted with an attenuated total reflection accessory and analyzed using the OPUS software (V.7.2).

**
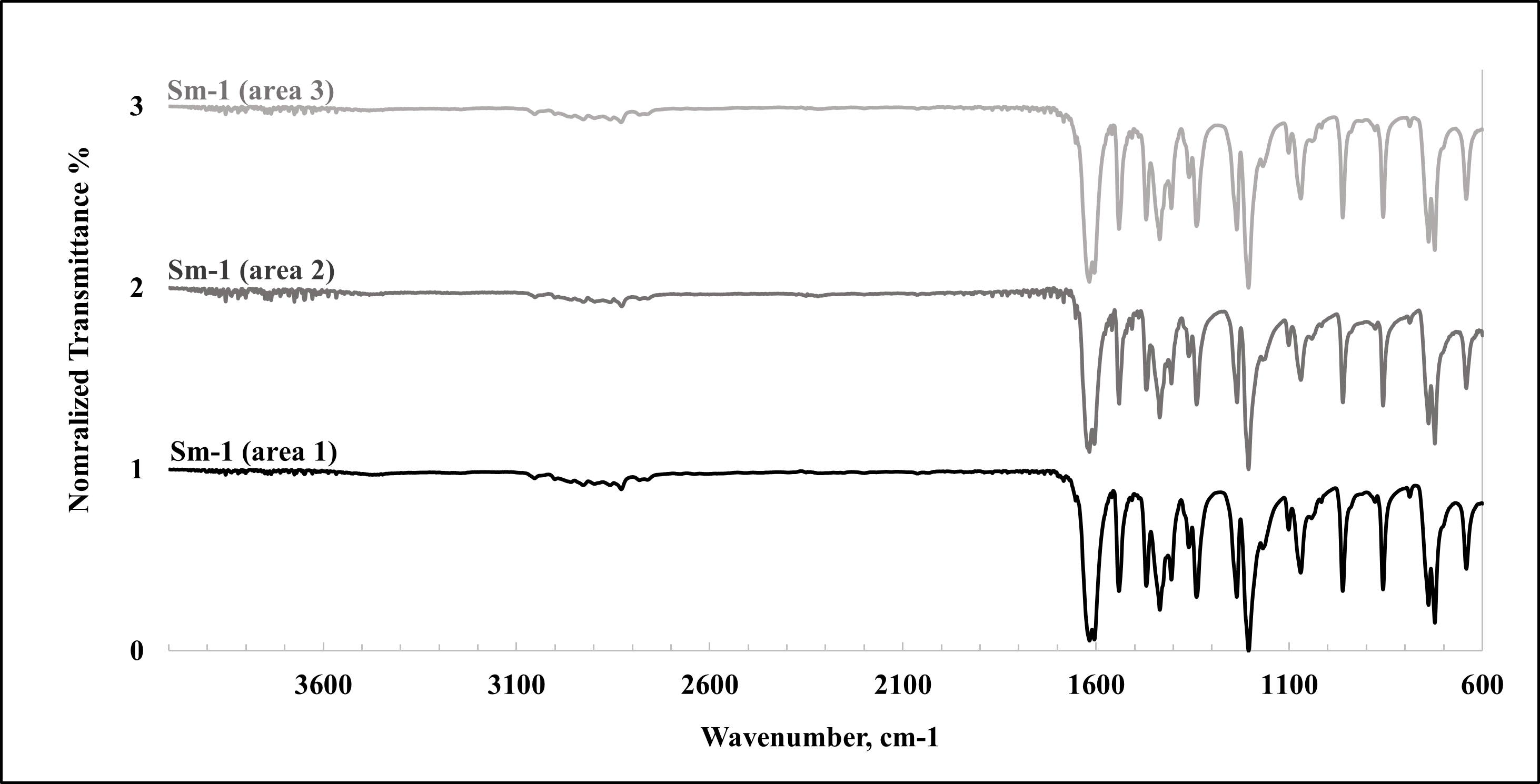
**

**Figure S3.** Bulk crystalline material of **Sm-1** was analyzed via FTIR in three areas to demonstrate homogeneity.


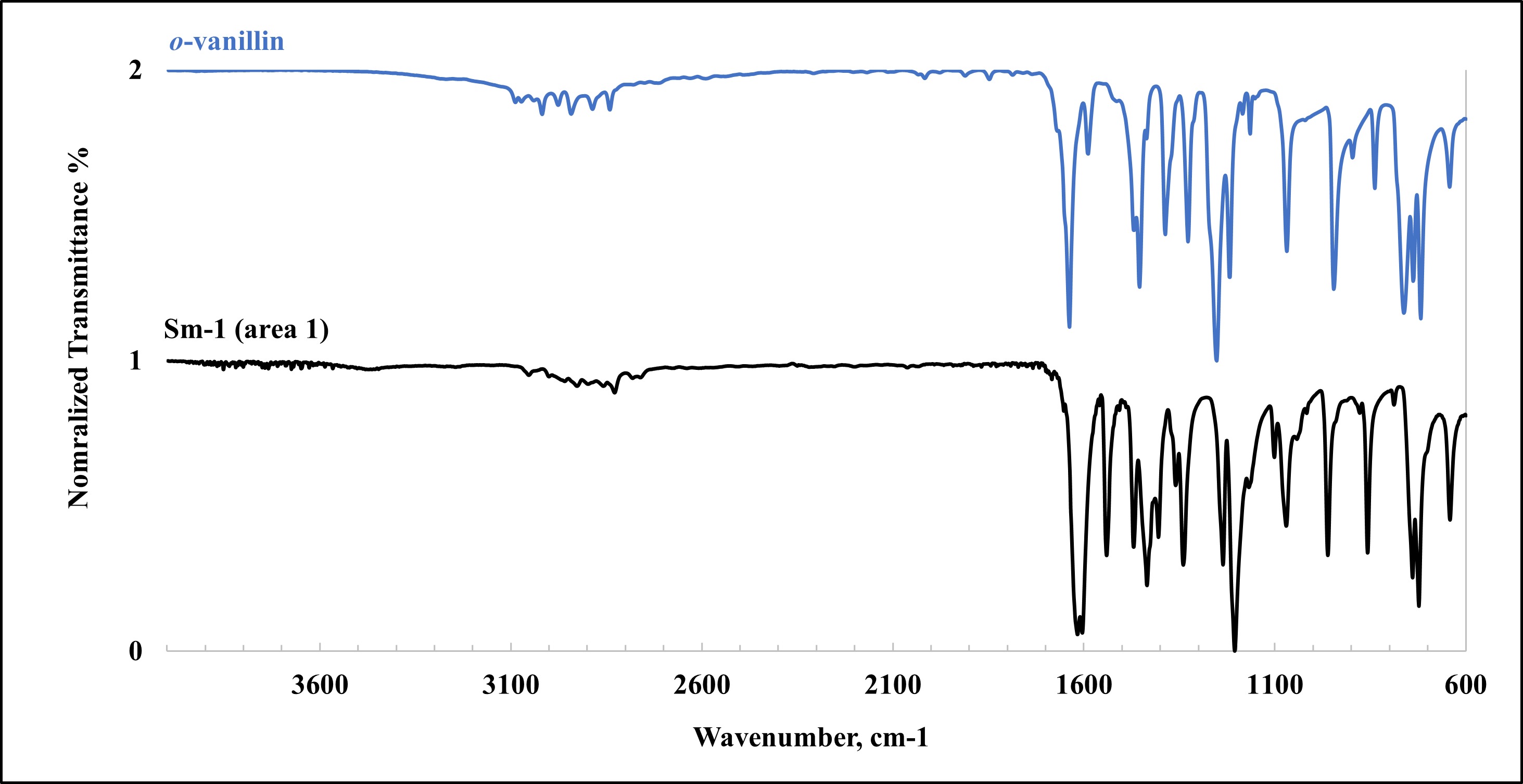


**Figure S4.** FTIR data of **Sm-1** and o-vanillin ligand.

**Diffuse Reflectance Spectroscopy**

The diffuse reflectance spectrum of **Sm-1** and *o*-vanillin were collected in ambient conditions on bulk crystalline material. An Ocean Optics Flame detector was coupled with a Mikropack DH-2000-BAL deuterium and halogen light source. Scattered light was collected with a fiber-optic cable, and spectra were referenced with BaSO_4_. Data were processed using OceanView spectroscopy software (V.2.0.8.). Compound **Sm-1** exhibits a strong absorption feature below 480 nm that originates from the *o*-vanillin ligand, as seen in Figure S5.

**
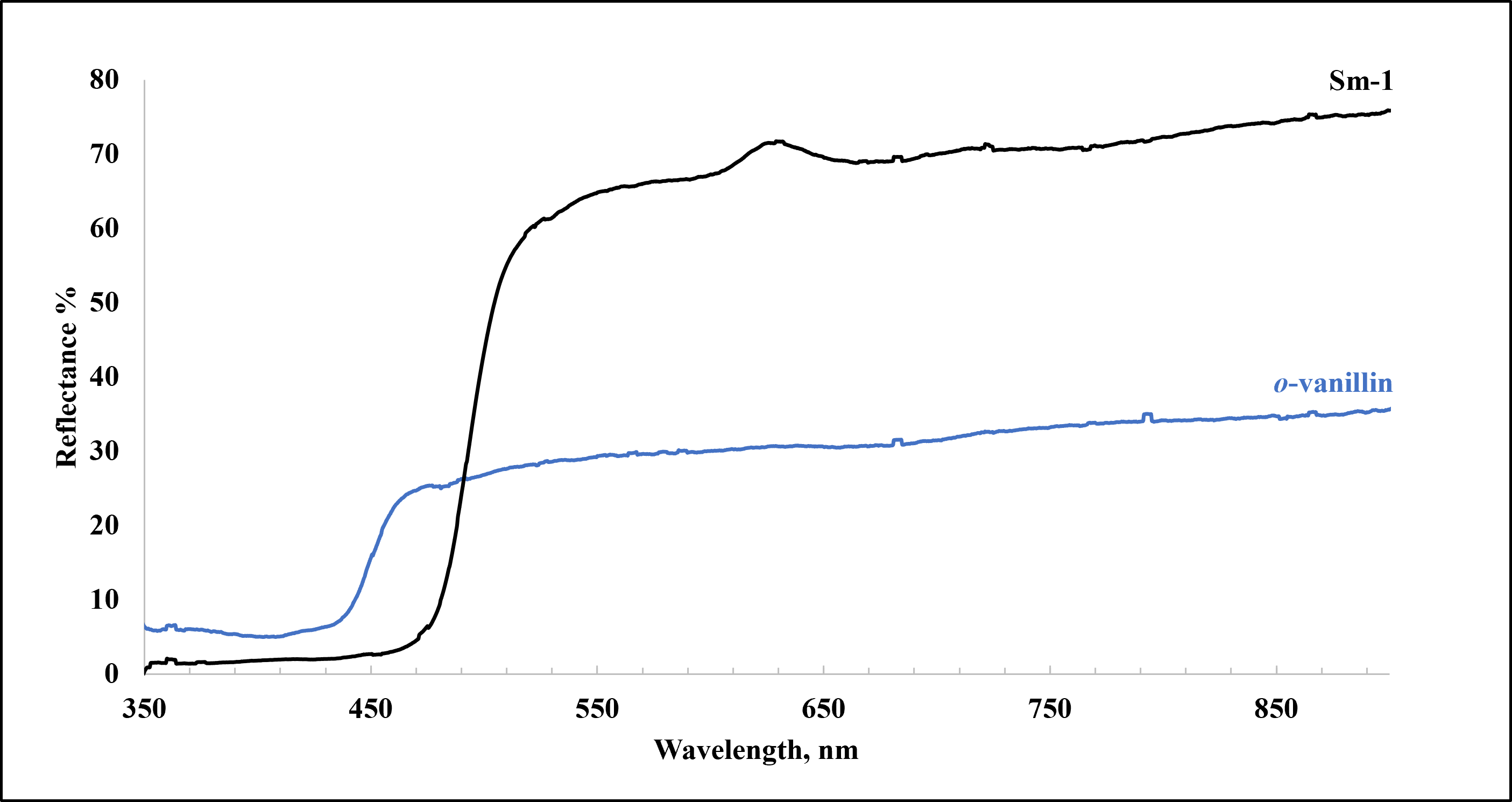
**

**Figure S5.** The solid-state diffuse reflectance spectra of **Sm-1** and *o*-vanillin**.**

**References**

Balachandran, V.; Parimala, K. (2012). *Spectrochim. Acta A Mol.* **95**, 354-368.

Mohamed, M. A.; Jaafar, J.; Ismail, A. F.; Othman M. H. D.; Rahman M. A. (2017). *Membrane Characterization*. **1**, 3-29.

Nandiyanto, A. B. D.; Oktiani, R.; Ragadhita, R. (2019). *Indones. J. Sci. Technol.* **4**(1), 97.
